# The Effect of Prior Gestational Diabetes on the Shape of the Glucose Response Curve during an Oral Glucose Tolerance Test 3 Years after Delivery

**DOI:** 10.1155/2020/4315806

**Published:** 2020-03-04

**Authors:** Timea Tänczer, Márk M. Svébis, Beatrix Domján, Viktor J. Horváth, Adam G. Tabák

**Affiliations:** ^1^Department of Internal Medicine and Oncology, Semmelweis University Faculty of Medicine, Budapest, Hungary; ^2^National Centre for Diabetes Care, Budapest, Hungary; ^3^School of Ph.D. Studies, Semmelweis University, Budapest, Hungary; ^4^Department of Public Health, Semmelweis University Faculty of Medicine, Budapest, Hungary; ^5^Department of Epidemiology & Public Health, University College London, London, UK

## Abstract

**Objective:**

Monophasic glucose response (MGR) during an oral glucose tolerance test (OGTT) and gestational diabetes mellitus (GDM) are predictors of type 2 diabetes mellitus (T2DM). We investigated the association between current MGR and (1) glucose tolerance during a pregnancy 3 years before and (2) current glucose tolerance status. We also sought (3) other determinants of MGR. *Research Design and Methods*. We conducted a nested case-control study of GDM (*n* = 47 early GDM, diagnosed between 16 and 20 weeks of gestation; *n* = 40 late GDM, diagnosed between 24 and 28 weeks of gestation) and matched healthy controls (*n* = 37, normal glucose tolerance during pregnancy) all free from diabetes at follow-up 3.4 ± 0.6 years after delivery. Glucose tolerance was determined by 2-hour 75 g OGTT. Monophasic and biphasic groups were defined based on serum glucose measurements during OGTT.

**Results:**

The biphasic group was younger, had lower triglyceride levels and area under the OGTT glucose curve, and was less frequently diagnosed with early GDM (25 vs. 45%, all *p* < 0.05). Women with a biphasic response also tended to have lower systolic blood pressure (*p* < 0.1). No differences were found in fasting and 2-hour glucose and insulin levels, or BMI. According to multiple logistic regression, MGR was associated with prior early GDM (OR 2.14, 95% CI 0.92-4.99) and elevated triglyceride levels (OR 2.28, 95% CI 1.03-5.03/log (mmol/l)).

**Conclusions:**

We found that more severe, early-onset GDM was an independent predictor of monophasic glucose response suggesting that monophasic response may represent an intermediate state between GDM and manifest type 2 diabetes.

## 1. Introduction

Fasting and 2-hour glucose levels during an oral glucose tolerance test (OGTT) are currently used for the diagnosis of diabetes and abnormal glucose tolerance [1]; however, the shape of the glucose response may provide further information on carbohydrate metabolism not used in clinical practice. The monophasic glucose response is related to insulin resistance, while people with more complex shapes have higher insulin sensitivity (IS) and lower risk for prediabetes and type 2 diabetes mellitus (T2DM) [2–5]. According to a prospective study, people with monophasic response during OGTT have a threefold increased risk for incident diabetes compared to those with more complex phenotypes [4].

Gestational diabetes is glucose intolerance diagnosed during pregnancy that is associated with a 7 times increased lifetime risk for T2DM compared to healthy controls [6, 7]. Guidelines recommend a follow-up of these women; however, the best method for the follow-up is elusive [8, 9]. Risk stratification of these women would be extremely important as diabetes development can be prevented or delayed in this population [10].

In the present study, we examined the association between the shape of current oral glucose response curves and (1) prior GDM (3 years before) and (2) current glucose tolerance status in a nested case-control study of women with prior GDM and controls. Furthermore, we looked for (3) other determinants of monophasic glucose response curves.

## 2. Materials and Methods

### 2.1. Setting

We report the results of a case-control study performed between 2008 and 2010 nested within the cohort of women who delivered at Saint Margit Hospital (Budapest, Hungary) between 01/January/2005 and 31/December/2006. This hospital serves a mostly urbanized population in central Hungary with a catchment population of 235,000 people [11].

A universal GDM screening was performed using a 3-step approach: (1) a fasting blood glucose measurement between 8 and 12 weeks of gestation to diagnose pregestational diabetes, (2) a 75 g OGTT between 16 and 20 weeks of gestation with the determination of fasting and 2-hour glucose, and (3) a second OGTT between 24 and 28 weeks of gestation. If any of the above tests were diagnostic of GDM, no further diagnostic tests were performed. Diagnostic cut-off values were based on the WHO 1999 criteria (fasting glucose ≥ 7 mmol/l and/or 2‐hour glucose level ≥ 7.8 mmol/l) [1].

Early GDM (*n* = 47) was defined as a diagnosis between 16 and 20 weeks of gestation and late GDM (*n* = 40) as a diagnosis between 24 and 28 weeks of gestation.

All GDM women received dietary advice and were encouraged to follow healthy lifestyles during pregnancy. If fasting and/or 1-hour postprandial glucose targets (<5.3 mmol/l and 7.0 mmol/l, respectively) were not achieved, insulin therapy was initiated according to the recommendation of the Hungarian Diabetes Association [9].

All GDM women and a randomly selected control group with normal glucose tolerance during pregnancy were invited for a follow-up investigation approximately 3.5 years after delivery. We excluded women with current diabetes mellitus (based on medication or current OGTT) and those with known diabetes before pregnancy.

The study protocol was approved by the Semmelweis University Regional and Institutional Committee of Science and Research Ethics (License number: 124/2007). All participants gave written informed consent before any study-related procedures were performed.

### 2.2. Participants

During the study period, altogether 3203 deliveries were recorded in Saint Margit Hospital. Thirteen women were excluded due to known pregestational diabetes and 45 women due to twin pregnancies. GDM was diagnosed in 193 cases (6.03%).

In this study, all GDM women (*n* = 193) as well as a randomly selected control group (*n* = 98) were investigated. All study participants were Caucasians. Of these potentially eligible women, 36 GDM and 8 control women were excluded due to current pregnancy, breastfeeding, or known diabetes.

**Figure 1:**
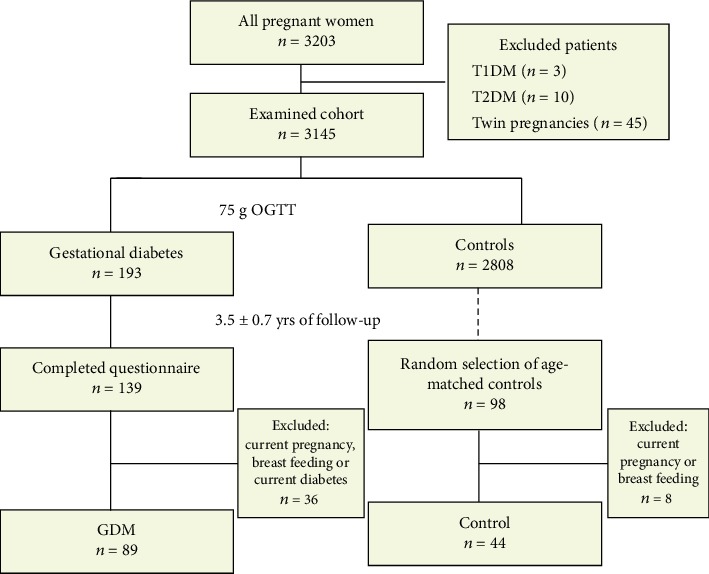
Flow chart of the study design.

For the purpose of the present analysis, we further excluded women who were diagnosed with diabetes mellitus based on their OGTT results at the follow-up investigation (all from the prior GDM group). Of all eligible women, data of 87/151 (56%) prior GDM and 37/90 (41%) controls were used (Figure 1).

### 2.3. Study Design

Three and a half years after delivery, participants were invited for a follow-up examination. Questionnaires were sent to all potentially eligible women (GDM *n* = 193, control women *n* = 98). Based on the information collected via these questionnaires, women who are currently pregnant, who are lactating, or who have known diabetes were excluded.

Eligible participants were invited to a detailed interview using a structured questionnaire on maternal sociodemographic characteristics and lifestyle habits (smoking, caffeine and alcohol consumption, physical activity, use of dietary supplements, and nutrition), as well as medical and reproductive history and family history of diabetes. In addition, a physical examination including anthropometrics and blood pressure was performed at this time.

Study participants were also tested for glucose tolerance (2-hour 75 g oral glucose tolerance test) with fasting blood samples taken for other laboratory parameters.

### 2.4. Covariates and Outcomes

Based on questionnaire data, age at follow-up, smoking status (coded as never, ex-, or current smoker (≥5 cigarettes/day)), known hypertension (doctor diagnosis or blood pressure-lowering medication use), and family history of diabetes (first-degree relative with diabetes) were determined.

Body weight was measured (rounded to the nearest 0.1 kg) in light clothing without shoes on a calibrated digital scale (Metripond plus BW 150, Metripond Kft., Hódmezővásárhely, Hungary). Height was measured without shoes in the Frankfort plane rounded to the closest centimeter. Body mass index (BMI) was calculated as weight (kg)/height (m)^2^. Waist circumference was measured in the midline between the iliac crest and the lowest point of the ribcage after a normal exhalation. Hip circumference was measured at the height of the greater trochanter. Both waist and hip were measured in the horizontal plane and rounded to the nearest centimeter.

Blood pressure (BP) was measured 3 times using a calibrated digital blood pressure meter (OMRON M4-I, Omron Electronics Kft., Budapest, Hungary) on the upper arm with an adequate-sized (to upper arm circumference) cuff after a 5-minute rest in sitting. The average of the second and the third measurements was used in further analysis. Hypertension was defined as a blood pressure ≥ 140/90 mmHg or doctor diagnosis of hypertension or the use of blood pressure-lowering medication.

### 2.5. Laboratory Measurements

All subjects underwent a standard 75 g OGTT with venous blood sampling for glucose at fasting and at 30, 60, 90, and 120 minutes after the glucose load. Other laboratory parameters were determined using the fasting sample.

The serum glucose level was measured using a glucose oxidase method on an AU 680 Beckman Chemistry System (Beckman Coulter Magyarország Kft., Budapest, Hungary); insulin was measured by the electrochemiluminescence immunoassay (ECLIA) on a Cobas e601 automated system (Roche Diagnostics Magyarország Kft., Budaörs, Hungary).

We determined HbA1c (high-performance liquid chromatography, Bio-Rad Magyarország Kft., Budapest, Hungary), serum lipids (cholesterol, high-density lipoprotein (HDL) cholesterol, low-density lipoprotein (LDL) cholesterol, and triglyceride), and C-reactive protein (CRP) on an AU 680 Beckman Chemistry System (Beckman Coulter Magyarország Kft., Budapest, Hungary).

### 2.6. Derived Variables

Based on the glucose values during the 2-hour OGTT, the following categories of glucose tolerance were defined: (i) diabetes mellitus (fasting plasma glucose ≥ 7.0and/or 2 h plasma glucose ≥ 11.1 mmol/l), (ii) impaired glucose tolerance (IGT) (fasting glucose < 7.0 mmol/l and 2 h glucose ≥ 7.8 and <11.1 mmol/l), (iii) impaired fasting glucose (IFG) (fasting glucose ≥ 6.1 and <7.0 mmol/l and 2 h glucose < 7.8 mmol/l), and (iv) normal glucose tolerance (NGT) (fasting glucose < 6.1 mmol/l and 2 h glucose < 7.8 mmol/l). In the present analysis, we combined the IFG and IGT groups as glucose intolerance (GI).

To estimate insulin resistance, we used the homeostasis model assessment (HOMA2 Calculator v.2.2, Diabetes Trials Unit, University of Oxford, Oxford, UK). Insulin sensitivity is characterized by HOMA2-S and *β*-cell function by HOMA2-B [12]. The individual area under the glucose curve (AUC_glu_) was calculated using trapezoidal integration.

“Monophasic shape” was diagnosed when plasma glucose showed one peak and followed an inverted U shape. “Biphasic shape” was diagnosed when the glucose curve reached a nadir after an initial increase and increased again until 120 min. A difference of ≥0.20 mmol/l between subsequent glucose values was considered clinically significant similarly to the definition used by Tschritter et al. [2].

### 2.7. Statistical Analysis

All analyses were conducted using SPSS 17.0 for Windows statistical package. Statistical significance was inferred at a two-sided *p* value < 0.05. Descriptives were presented as the arithmetic mean ± SD, estimate, SE, or median [interquartile range] for continuous variables and as percentages for categorical variables.

To compare women with monophasic and biphasic curves, we used independent sample *t*-tests (continuous variables) and chi-square or Fischer exact tests (categorical variables) as appropriate. The distribution of continuous variables was tested for normality, and variables with a skewed distribution were log transformed.

Logistic regression was used to analyse the relationship between severity of GDM and monophasic glucose curve. Univariate tests were used to identify parameters that were associated with monophasic glucose curves (*p* < 0.1). Parameters associated univariately with monophasic glucose curves were added to a logistic regression model. Backward stepwise elimination was used to define independent predictors of the monophasic shape.

## 3. Results

### 3.1. Baseline Characteristics

In total, 87 women with prior GDM and 37 age-matched controls with normal glucose tolerance during pregnancy were examined. Descriptive characteristics of participants divided by GDM status are presented in Table 1. Of the participants, 38% (*n* = 47) had early GDM, 32% (*n* = 40) had late GDM, and 30% (*n* = 37) had normal glucose tolerance during pregnancy. Prior GDM women were older (mean difference (MD), SE: 1.74, 0.78 years) and had higher fasting glucose (1.96, 0.08 mmol/l) and 2-hour glucose (1.29, 0.30 mmol/l), higher A1C (1.52, 0.06%), and higher areas under the glucose curve (logAUC_glu_, 2.07, 0.38). At follow-up 3.5 years after delivery, glucose intolerance (GI) was found in 24 women (19.4%; *n* = 18 with IGT, *n* = 6 with IFG), all but one in the prior GDM group. More women had hypertension (21.8% vs. 5.4%) in the prior GDM group, and they had higher systolic (MD, SE: 8.0, 2.9 mmHg) and diastolic (7.9, 1.93 mmHg) blood pressure values. There was no statistically significant difference between the two groups in the other examined metabolic parameters (serum lipids and fasting insulin levels).

### 3.2. Parameters Potentially Associated with a Monophasic OGTT Response

**Figure 2:**
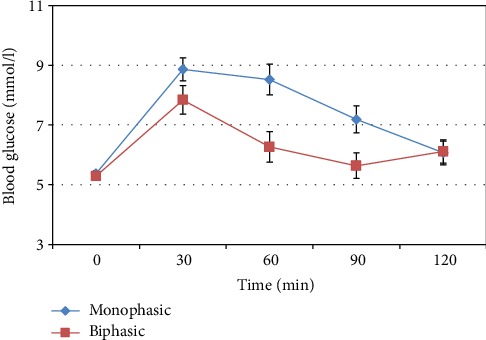
Average glucose levels (and 95% confidence intervals) during OGTT by the shape of the glucose response curve.

Women in the monophasic group were older. However, there was no difference between groups in body mass index, fasting glucose and insulin, 2-hour glucose, A1C levels, or the frequency of glucose intolerance. The areas under the glucose (AUC_glu_) curves were significantly higher in the monophasic group, and the time of the glucose peak was later (more frequently at 60 minutes or later: 37.8 vs. 20.5%, *p* = 0.035) (Table 2). Moreover, all glucose values during the 75 g OGTT were significantly higher in the monophasic group compared to the biphasic group except for the fasting and 2-hour glucose levels (Figure 2).

More women were diagnosed with early GDM in the monophasic group, and there was a nonsignificantly higher prevalence of multiparity among them. There was no difference in any of the other investigated parameters, except for elevated levels of triglycerides in the monophasic group (Table 2).

### 3.3. Pregnancy Predictors of the Monophasic Glucose Curve

Among variables assessed during or immediately after pregnancy, older age at delivery and early-onset GDM were independent predictors of an increased risk of a monophasic response (Table 3).

### 3.4. Independent Determinants of the Monophasic Glucose Curve

When variables assessed at follow-up were also available for the model, early-onset GDM remained an independent predictor with higher triglyceride levels measured at follow-up (Table 4).

## 4. Discussion

In our case-control study embedded in a population-based cohort of deliveries, we found that older age at delivery and early-onset GDM were independent predictors of the monophasic glucose response 3.5 years after delivery. Furthermore, early-onset GDM remained an independent predictor of the monophasic response even after taking into account metabolic parameters measured at follow-up.

Previously, several studies examined the cross-sectional relationship between monophasic glucose curves and parameters characterizing insulin sensitivity (fasting glucose and insulin levels, disposition index, AUC_glu_, and AUC_ins_) and *β*-cell function, indicating better glucose tolerance in the biphasic group in nonpregnant people [2, 13–15]. The monophasic phenotype was also associated with higher AUC_glu_ among healthy children, teenagers, and adults with normal glucose tolerance [2, 13–17]. In our study, we confirmed that the monophasic shape was associated with higher AUC_glu_ while the fasting and 2-hour glucose values were similar.

People with OGTTs that have a more complex shape have higher insulin sensitivity and a lower risk for prediabetes and type 2 diabetes [15]. According to a cohort of 2445 Caucasian nondiabetic subjects, the conversion rate to type 2 diabetes was twice as high in the monophasic group compared to the biphasic group with 7-8 years of follow-up [4]. In a study using 3-hour 7-point OGTTs in women with a history of previous GDM or uncomplicated pregnancy, the more complex shape of the OGTT glucose response was related to better insulin sensitivity with the monophasic shape having the worst insulin resistance and the highest risk of type 2 diabetes [5].

In addition to lower hepatic and peripheral insulin sensitivity in vivo, the monophasic group has inadequate compensation in first- and second-phase insulin secretion, measured as impaired *β*-cell function independently of fasting and 2-hour glucose and insulin levels, probably secondary to lipotoxicity [18].

We found that time to glucose peak during the OGTT was different between monophasic and biphasic subjects: the former group reached their peak more frequently at 60 minutes or later compared to the biphasic group. Similar results were obtained in a postpartum follow-up study of GDM women: for those women without glucose intolerance at follow-up (nonprogressors), the OGTT glucose peaked at 30 minutes, while for progressors, it peaked at 60 minutes. Furthermore, even among women with normal glucose tolerance, both increased AUC_glu_ and a delayed glucose peak predicted the development of type 2 diabetes by identifying people with an early abnormality of *β*-cell function [17]. In addition to increased diabetes risk, later glucose peak was also associated with an increased risk of cardiovascular mortality in a Danish register [15]. According to a study with repeated OGTTs, the time to glucose peak has acceptable reproducibility compared to other novel measures of mild glucose intolerance [18].

Though some studies have found a relationship between monophasic glucose response and an increased BMI [5, 13, 14], we did not find such a difference in different measures of obesity (waist-hip ratio and BMI). Possible explanations for these differences could be related to the characteristics of the investigated populations: some of these studies also included men and the age distribution is also different between studies. The difference could also be a power issue explained by the relatively small sample size of our investigation.

Prior GDM is a strong risk factor for metabolic syndrome with some of its components present already before glucose intolerance develops [19, 20]. It is particularly interesting that higher triglyceride levels were cross-sectionally associated with the monophasic curve independent of prior early-onset GDM status in our study.

Our study has some limitation that has to be acknowledged: the relatively small sample size could decrease statistical power leading to omission of potentially true associations (type II error). The single OGTT has poor reproducibility which could lead to misclassification, further limiting study power [18]. It should be mentioned that the shape of the glucose curve has high reproducibility even over a 3-year follow-up [21]. The generalization of our results is also limited by the fact that only Caucasians participated in the study. Potential gestational predictors were not systematically collected; thus, important predictors may be omitted. No further follow-up after the OGTT was done; thus, our conclusion regarding the association between the monophasic glucose curve and an increased diabetes risk is based on literature analogues [22].

The strengths of this study relate to its nested case-control design: despite the small sample size, our participants (both cases and controls) well represent the pregnant population in Saint Margit Hospital. It must be emphasized that study participants were highly phenotyped at follow-up using gold standard measures.

In conclusion, we found that women with early-onset GDM more frequently have a monophasic glucose response during an OGTT that could be an early marker of glucose intolerance following a pregnancy complicated by GDM even without elevated fasting or 2-hour glucose values. By extrapolating data from the literature on the natural history of type 2 diabetes, it is conceivable that the monophasic blood glucose curve represents an intermediary state between GDM and later type 2 diabetes, metabolic syndrome, and cardiovascular disease. It is also plausible that other measures of the shape of the glucose curves obtained during the OGTT could be early indicators of *β*-cell dysfunction and diabetes in high-risk subjects earlier than fasting or postload blood glucose values.

## Figures and Tables

**Table 1:** Baseline characteristics of study participants by prior glucose tolerance.

Participants (*n*)	GDM (87)	Control (37)	*p*
Age at follow-up (year)	35.7 ± 3.9	34.0 ± 4.1	0.03
Follow-up (year)	3.4 ± 0.6	3.4 ± 0.3	0.84
Weight (kg)	68.7 ± 13.9	67.1 ± 12.5	0.52
BMI (kg/m^2^)	25.5 ± 4.9	24.3 ± 4.5	0.19
Waist circumference (cm)	84.2 ± 11.6	81.0 ± 9.1	0.12
Waist-to-hip ratio	0.82 ± 0.07	0.79 ± 0.06	0.08
Smoking, *n* (%)	19 (21.8)	8 (21.6)	0.98
Number of previous pregnancies, *n* (%)			
None	32 (37.2)	13 (36.1)	0.141
One	22 (25.6)	15 (41.7)	
More than one	32 (37.2)	8 (22.2)	
Fasting glucose (mmol/l)	5.4 ± 0.4	5.2 ± 0.4	0.017
120 min glucose (mmol/l)	6.5 ± 1.6	5.2 ± 1.3	<0.0001
Glucose intolerance, *n* (%)	23 (26.4)	1 (2.7)	0.001
A1C (%)	5.5 ± 0.3	5.4 ± 0.3	0.02
AUC_glu_ (mmol/l·min)^∗^	[906.8]	[691.5]	<0.0001
Fasting insulin (*μ*U/ml)^∗^	[9.5]	[8.1]	0.20
HOMA2-S^∗^	[78.7]	[95.5]	0.08
HOMA2-B^∗^	[96.9]	[90.45]	0.61
HDL cholesterol (mmol/l)	1.5 ± 0.3	1.5 ± 0.2	0.57
LDL cholesterol (mmol/l)	2.9 ± 0.7	2.8 ± 0.7	0.53
Triglycerides (mmol/l)^∗^	[1.0]	[0.9]	0.37
Systolic blood pressure (mmHg)	121 ± 15	113 ± 13	0.005
Diastolic blood pressure (mmHg)	78 ± 10	70 ± 9	0.00
Hypertension, *n* (%)	19 (21.8)	2 (5.4)	0.03
C-reactive protein (mg/l)^∗^	[1.1]	[0.95]	0.25

Mean ± SD or ∗ = median [IQR].

**Table 2:** Characteristics of participants by glucose curve phenotype.

Shape of glucose curve	Biphasic	Monophasic	*p*
Age at follow-up (year)	34.2 ± 4.0	35.7 ± 3.9	0.04
Follow-up (year)	3.4 ± 0.7	3.4 ± 0.6	0.96
Weight (kg)	65.7 ± 11.8	69.6 ± 14.2	0.1
BMI (kg/m^2^)	24.3 ± 4.0	25.6 ± 5.0	0.15
Waist circumference (cm)	81.4 ± 9.6	84.3 ± 11.5	0.14
Waist-to-hip ratio	0.81 ± 0.06	0.81 ± 0.07	0.78
Smoking, *n* (%)	12 (27.3)	15 (18.8)	0.36
Number of previous pregnancies, *n* (%)			0.06
None	21 (48.8)	24 (30.4)	
One	11 (25.6)	26 (32.9)	
More than one	11 (25.6)	29 (36.7)	
Early GDM, *n* (%)^∗^	11 (23.4)	36 (45.0)	0.034
Fasting glucose (mmol/l)	5.3 ± 0.4	5.4 ± 0.5	0.26
2-hour glucose (mmol/l)	6.1 ± 1.3	6.1 ± 1.3	0.873
Glucose intolerance, *n* (%)	6 (14)	18 (23)	0.34
A1C (%)	5.5 ± 0.3	5.5 ± 0.3	0.46
AUC_glu_ (mmol/l∗min)^∗^	753 [129]	897 [310]	0.009
Fasting insulin (*μ*U/ml)^∗^	8.47 [4.38]	9.07 [9.83]	0.66
HOMA2-S^∗^	90.0 [44.7]	82.2 [89.4]	0.82
HOMA2-B^∗^	98.0 [44.9]	91.3 [56.1]	0.90
HDL cholesterol (mmol/l)	1.5 ± 0.3	1.5 ± 0.3	0.21
LDL cholesterol (mmol/l)	2.8 ± 0.7	2.9 ± 0.7	0.44
Serum triglyceride (mmol/l)^∗^	0.9 [0.8]	1.0 [1.0]	0.033
Systolic blood pressure (mmHg)	116 ± 13	121 ± 16	0.06
Diastolic blood pressure (mmHg)	74 ± 10	77 ± 10	0.17
Hypertension, *n* (%)	5 (12)	16 (20)	0.32
C-reactive protein (mg/l)^∗^	1.1 [2.05]	1.1 [2.95]	0.25

Mean ± SD or ∗ = median [IQR].

**Table 3:** Predictors of a monophasic glucose response at follow-up assessed during the index pregnancy.

	OR	95% CI	*p*
Age at delivery (year)	1.1	0.99-1.21	0.09
Early-onset GDM	2.56	1.01-5.96	0.03

Other parameters available for the model: number of previous pregnancies.

**Table 4:** Independent determinants of a monophasic glucose response assessed during the index pregnancy and at follow-up.

Covariate	OR	95% CI	*p*
Early-onset GDM	2.14	0.92-4.99	0.07
Serum triglyceride (log(mmol/l))	2.28	1.03-5.03	0.04

Other parameters available for the model: waist circumference, age, systolic blood pressure, and age at delivery.

## Data Availability

All participant information will be available after de-identification. And data could be shared with researchers who provide a methodologically sound purpose. Statistical methods and the calculations (SPSS output files) will be available for special request as well. Previously reported data were used to support this study and are available at doi: 10.1016/j.jcjd.2017.01.003. In this prior study, we used the identical study cohort to prove a different hypothesis. Proposals should be directed to tabak.adam@med semmelweis-univ.hu. to gain access. Data requestors will need to sign a data access agreement.

## Abbreviation

A1C: Glycated hemoglobin A1c
AUC: Area under the plasma concentration-time curve
BMI: Body mass index
BP: Blood pressure
BUN: Blood urea nitrogen
CRP: C-reactive protein
*γ*GT: Gamma-glutamyl transferase
GI: Glucose intolerance
GDM: Gestational diabetes mellitus
HDL-C: High-density lipoprotein cholesterol
HOMA: Homeostasis model assessment
HOMA2-S: HOMA insulin sensitivity
HOMA2-B: HOMA insulin secretion
IFG: Impaired fasting glucose
IGT: Impaired glucose tolerance
LDL-C: Low-density lipoprotein cholesterol
NGT: Normal glucose tolerance
OGTT: Oral glucose tolerance test
SD: Standard deviation
T2DM: Type 2 diabetes mellitus
TC: Total cholesterol
TG: Triglyceride.

## Additional Points

*Key Messages*. Gestational diabetes (GDM) is associated with an increased lifetime risk of type 2 diabetes mellitus (T2DM). The monophasic glucose shape during OGTT is related to higher risk for diabetes. Early-onset GDM was an independent predictor of the monophasic phenotype suggesting that monophasic response may represent an intermediate state between GDM and manifest T2DM. *Strengths and Limitations of This Study*. (i) A nested case-control study was performed to examine the association between glucose response curves and gestational diabetes. (ii) Despite the small sample size, our participants (both cases and controls) well represent the pregnant population from a Hungarian urban hospital. It must be emphasized that study participants were highly phenotyped at follow-up using gold standard measures. (iii) A further limitation of our study is the relatively small sample size, which could decrease statistical power leading to omission of potentially true associations. (iv) The single OGTT has poor reproducibility which could lead to misclassification, further limiting study power. (v) Potential gestational predictors were not systematically collected; thus, important predictors may have been omitted.
